# „Brain sagging dementia“ – eine seltene, potenziell reversible Demenzursache

**DOI:** 10.1007/s00115-025-01803-z

**Published:** 2025-01-22

**Authors:** V. Sondermann, J. C. Gerber, W. H. Polanski, M. Brandt, J. Schäfer, E. Dinter, R. Haußmann

**Affiliations:** 1 Klinik und Poliklinik für Neurologie, Universitätsklinikum Carl Gustav Carus an der Technischen Universität Dresden, Fetscherstr. 74, 01307 Dresden, Deutschland; 2 Institut und Poliklinik für Diagnostische und Interventionelle Neuroradiologie, Universitätsklinikum Carl Gustav Carus an der Technischen Universität Dresden und Medizinische Fakultät der Technischen Universität Dresden, Fetscherstr. 74, 01307 Dresden, Deutschland; 3 Klinik und Poliklinik für Neurochirurgie, Universitätsklinikum Carl Gustav Carus an der Technischen Universität Dresden, Fetscherstr. 74, 01307 Dresden, Deutschland; 4 Klinik für Neurologie und Geriatrie, Elblandklinikum Meißen, Nassauweg 7, 01662 Meißen, Deutschland; 5 Klinik und Poliklinik für Psychiatrie und Psychotherapie, Universitätsklinikum Carl Gustav Carus an der Technischen Universität Dresden, Fetscherstr. 74, 01307 Dresden, Deutschland; 6 DZNE, Deutsches Zentrum für Neurodegenerative Erkrankungen, Dresden, Deutschland; 7 Klinik und Poliklinik für Psychiatrie und Psychotherapie, Universitäts DemenzCentrum UDC, Universitätsklinikum Carl Gustav Carus an der Technischen Universität Dresden, Fiedlerstraße 74, 01307 Dresden, Deutschland

## Anamnese

Ein 59-jähriger Mann stellte sich mit seit sechs Monaten langsam progredienter Gedächtnisstörung mit leichtgradig demenziellem Syndrom vor. Zusätzlich wurden Kopfschmerzen seit einigen Monaten, eine Antriebsminderung und ein erhöhtes Schlafbedürfnis berichtet. Die Kopfschmerzen wurden als zunehmend unter körperlicher Belastung und im Tagesverlauf beschrieben. Darüber hinaus wurde fremdanamnestisch ein innerhalb der letzten Monate progredient disinhibiertes Verhalten berichtet. An relevanten Vorerkrankungen bestand ein Z. n. Germinom 1993 mit stereotaktischer Probenentnahme und Einlage eines Iod125-Seeds rechts thalamisch mit residuellen Doppelbildern beim Blick nach rechts oben ohne darüberhinausgehende fokal-neurologische Defizite. Die Familienanamnese für neurodegenerative Erkrankungen war leer.

Die Vorstellung erfolgte nach externer Diagnostik (cMRT und Lumbalpunktion). Dabei wurden neben dem einliegenden Seed keine globalen oder fokalen Atrophiezeichen beschrieben. Bei leichtgradig erhöhtem Gesamt- und Phospho-Tau war die Verdachtsdiagnose einer Alzheimer-Demenz gestellt und die Vorstellung in unserem Zentrum veranlasst worden. Wir ergänzten eine differenzierende neuropsychologische Testung. Hier fielen Defizite in den mnestischen und exekutiven Domänen sowie Disinhibitionszeichen auf, die sich auch in der Frontal Assessment Battery (FAB) objektivieren ließen. Bei differenzialdiagnostisch zu diskutierender frontotemporaler Demenz erfolgte eine erneute Liquorpunktion. Diese zeigte eine normwertige Abeta-Ratio, ein erhöhtes Phospho- und Gesamt-Tau bei normwertigem Neurofilament ohne Zellzahl- oder Eiweißerhöhung. In der interdisziplinären Fallbesprechung mit den Kollegen der Neuroradiologie inkl. Sichtung der externen MRT wurde eine Herniation des temporomesialen Hirnparenchyms beidseits mit Einengung der Cisterna ambiens beschrieben. Ein meningeales Enhancement, ein Kleinhirntonsillentiefstand oder subdurale Hygrome bestanden nicht. Weiterhin wurde ein minimaler nicht raumfordernder Parenchymdefekt thalamisch bis mesenzephal, den Iod125-Seed umgebend, dargestellt.

Der Patient wurde bei Verdacht auf ein zum Zeitpunkt der initialen Bildgebung, also präpunktionell, bestehendes Liquorunterdrucksyndrom zur weiteren Diagnostik und Therapie stationär aufgenommen. In der klinischen Untersuchung zeigte sich eine frontale Disinhibition. Es bestand weiter eine diffuse, im Tagesverlauf zunehmende Kopfschmerzsymptomatik ohne typische orthostatische Komponente.

Die aktualisierte und erweiterte Bildgebung mittels kranialer und spinaler MRT zeigte einen stabilen Befund ohne weitere bildgebende Merkmale einer intrakraniellen Hypotension. In externen CT-Aufnahmen aus dem Jahr 2013 war die Kaudalverlagerung der Mittellinienstrukturen noch nicht zu sehen, sodass ein anlagebedingter oder direkter Effekt durch die 1993 erfolgte Seed-Einlage unwahrscheinlich erschien.

## Befunde

**Abb. 1:**
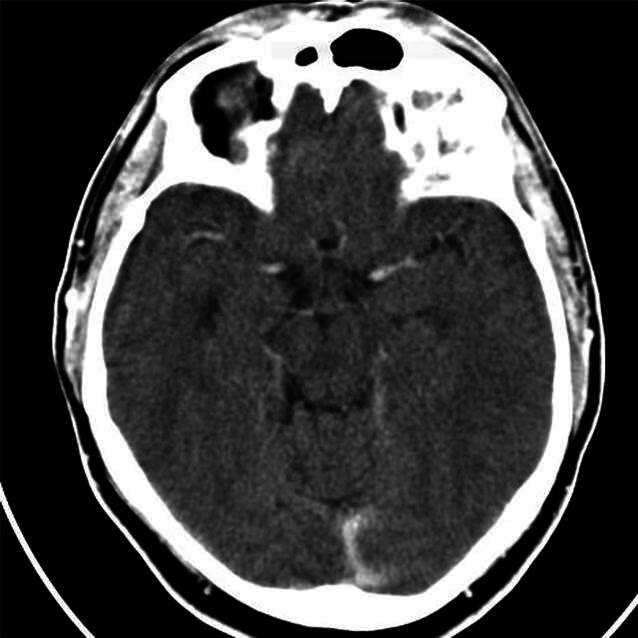
cCT 2013. Mittellinienstrukturen ohne Sagging; Hippokampusstiele, soweit beurteilbar, nicht herniert

Die bildgebenden Befunde sowie die Ergebnisse der neuropsychologischen Testung sind Abb. 1 und 2 sowie Tab. 1 zu entnehmen.

**Abb. 2:**
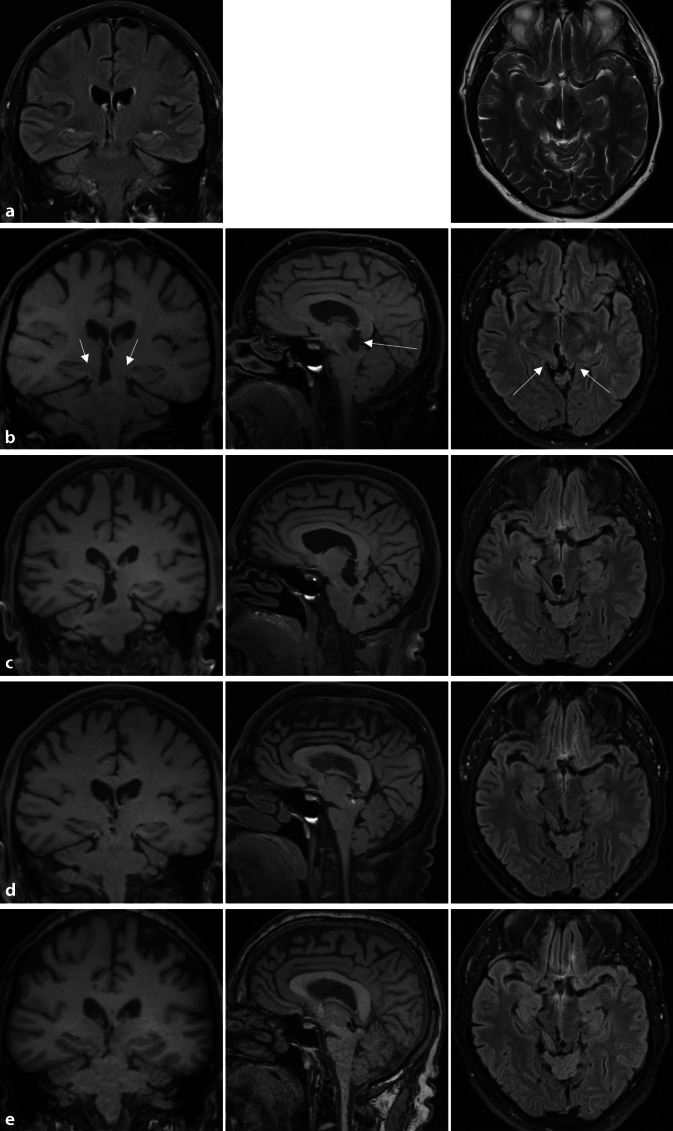
Kraniale Bildgebungen (MRT) im zeitlichen Verlauf: exemplarische cMRT-Bildgebungen koronar (*erste Spalte*), sagittal (*zweite Spalte*) und transversal (*dritte Spalte*). **a** Externe Bildgebung, 08/2023 (koronar FLAIR, axial T2): Kaudalverlagerung der Mittellinienstrukturen, kleiner zystischer Defekt mit Flüssigkeitsansammlung um den ehemaligen Iod125-Seed, Hernierung des Hippokampusgewebes. **b** 11/2023, T1 koronar: weiterhin Kaudalverlagerung der Mittellinienstrukturen, Hernierung der temporomesialen Strukturen beidseits (*exemplarisch Pfeile*). T1 sagittal: zunehmende zystische Läsion mesenzephal (*Pfeil*), Kaudalverlagerung Pontomesenzephalon, Kleinhirntonsillentiefstand, T2 FLAIR transversal: zystische Läsion mesenzephal, Hernierung der Hippokampusstiele am Tentorium cerebelli (*exemplarisch Pfeile*). **c** 03/2024: Größenzunahme der mesenzephalen zystischen Läsion mit Verdrängung des Pons bei weiter bestehender Kaudalverlagerung der Mittellinienstrukturen. **d** 05/2024, T1 koronar/sagittal sowie T2 FLAIR transversal: nach Anlage des Ommaya-Reservoirs deutlich regrediente zystische Läsion mesenzephal, weiterhin Kaudalverlagerung der Mittellinienstrukturen mit Herniation der Hippokampusstiele bds. **e** 11/2024, MPRAGE koronar/saggital sowie T2 FLAIR transversal: im Vergleich zu Mai 2024 unveränderter Befund

**Tab. 1:** Testpsychometrische Befunde im zeitlichen Verlauf

**Testverfahren**	**Ergebnisse **(im Vergleich zur Altersnorm) ↓ = unterdurchschnittlich ● = durchschnittlich ↑ = überdurchschnittlich				
	**Normwert**	**Verlauf**			
MWT‑A (kristalline Intelligenz)		●(03.02.2024)			
LPS-UT3 (fluide Intelligenz)		●(03.02.2024)			
		20**23/12**	20**24/02**	20**24/05**	20**24/11**
*MMST*	Punkte (von 30)	–	27	28	28
*MoCA*	Punkte (von 30)	22	–	–	–
*FAB‑D*	Punkte (von 18)	–	13	17	12
*CERAD: Wortliste*	Lernen	(z: −1,7)*	z: −2,2	z: 0,1	z: −1,5
	Freier Abruf (verzögert)	(z: −3,8)*	z: −3,8	z: −2,1	z: −2,6
	Wiedererkennen	(z: −3,3)*	z: −2,9	z: 0,8	z: −1,5
*WMS-R:*	Kurzfristig	–	PR: 13	PR: 8	PR: 13
*Visuelle Wiedergabe*	Längerfristig	–	PR: 0	PR: 0	PR: 0
*WMS-R:*	Akustische Merkspanne	–	PR: 20	PR: 48	PR: 67
*Zahlenspanne*	Akustisches Arbeitsgedächtnis	–	PR: 38	PR: 67	PR: 38
*AAT: Benennen (Gegenstände)*	Einfache Nomina	–	30/30	30/30	30/30
	Nomina Komposita	–	30/30	30/30	30/30
*CERAD: verbale Flüssigkeit*					
Anzahl Tiere	Semantische Flüssigkeit	–	z: −1,1	z: 0,1	z: −0,7
Anzahl S‑Wörter	Phonematische Flüssigkeit	–	z: −1,6	z: −0,7	z: −2,2
*TMT A*	Geschwindigkeit	(z: 2,0)*	z: 1,1	z: 1,2	z: 1,8
*TMT B*	Geteilte Aufmerksamkeit	(z: 0,4)*	z: −0,9	z: −0,7	z: −0,4
*NAI:*					
Labyrinth-Test	Planungsfähigkeit	(C: 8)*	C: 5	C: 6,5	C: 6,5
Farb-Wort-Test	Kognitive Umstellfähigkeit	(C: 3,5)*	C: 4,5	C: 4	C: 5

Ergebnisse angegeben mit Standardabweichung (z), Prozentrang (PR) oder C‑Score Dezile (C), jeweils zur Alters- und Bildungsnorm

*MWT‑A* Mehrfachwahl-Wortschatz-Intelligenztest, *LPS-UT3* Leistungsprüfsystem Untertest 3, *MMST* Mini-Mental-Status-Test, *MoCA* Montreal Cognitive Assessment, *FAB‑D* Frontal Assessment Battery, deutsche Version, *CERAD* Consortium to Establish a Registry for Alzheimer’s Disease Testbatterie, *WMS‑R* Wechsler Memory Scale, revidierte Fassung, *AAT* Aachener Aphasietest, *TMT* Trail Making Test

*Testung in anderer Abteilung

## Diagnose

In der Zusammenschau bestand die Verdachtsdiagnose einer „brain sagging dementia“ (BSD) mit typischen mnestischen Defiziten und begleitender Disinhibition in Folge eines bislang nicht lokalisierbaren Liquorlecks bzw. einer Liquorfistel.

## Therapie und Verlauf

Es erfolgte ein lumbaler Blutpatch. Danach zeigte sich kurzfristig keine klinische Befundänderung, sodass zunächst Verlaufskontrollen vereinbart wurden. In der Verlaufsbildgebung nach einem Monat (nicht dargestellt) wurde eine unveränderte Kaudalverlagerung der Mittellinienstrukturen sowie eine Größenzunahme der mesenzephalen liquorgefüllten Struktur um den ehemaligen rechts thalamischen Seed bei Z. n. Germinom festgestellt. Diese Zyste zeigte sich innerhalb eines weiteren Monats (Abb. 2c) weiter größenprogredient mit zunehmender Kaudalverlagerung der di- und pontomesenzephalen Mittellinienstrukturen und Kompression des Mesenzephalons. Neben einer Gangunsicherheit mit ataktischer Komponente bestanden nun eine inkomplette supranukleäre vertikale Blickparese, ständiger Reizhusten, Kopfschmerzen mit täglichem Schmerzmittelbedarf sowie eine ausgeprägte Affektlabilität.

Aufgrund des progredienten klinischen und bildmorphologischen Befundes erfolgte nach interdisziplinärer Falldiskussion eine neurochirurgische Intervention in Form einer stereotaktischen Anlage eines Ommaya-Reservoirs in die Zyste. Die postoperativen Kontrollen zeigten eine komplett regrediente zystische Struktur und entfaltete mesenzephale und pontine Strukturen bei jedoch weiterhin herniertem Hippokampusgewebe beidseits. Klinisch waren der Antrieb und Gangunsicherheit gebessert bei regredientem Kopfschmerzsyndrom.

In der testpsychometrischen Kontrolle sechs Wochen nach Operation wurden eine Regredienz von Disinhibition und exekutiver Dysfunktion und eine deutliche Besserung mnestischer Defizite, bei querschnittlich aber weiterhin defizitären Gedächtnisleistungen objektiviert. Die supranukleäre Blickparese war vollständig regredient.

Im Rahmen einer Verlaufsuntersuchung nach sechs weiteren Monaten wurde erneut eine progrediente Verschlechterung mit Zunahme der initialen Symptomatik, insbesondere in kognitiver Hinsicht, beschrieben. Testpsychometrisch zeigte sich eine erneute Verschlechterung der mnestischen Defizite. Auch die behavioralen Auffälligkeiten bestanden wieder auf präinterventionellem Niveau. Klinisch wurden zudem Schluckstörungen, ein gehäuftes Gähnen sowie ein zunehmender Hustenreiz berichtet. Nach körperlicher Aktivität und längerem Gehen imponierte das Gangbild zunehmend ataktisch.

Bildgebend wurde eine nach Entlastung komplett regrediente mesenzephale Zyste sowie ein konstantes „brain sagging“ mit Kaudalverlagerung okzipitotemporalen Hirnparenchyms und des Hirnstamms sowie rechtsbetonte Herniation der Hippocampi beidseits dargestellt.

Es erfolgte die erneute spinale Bildgebung mittels MRT. Darüber hinaus wurde bei klinisch und anhand des intrakraniellen Befundes weiterhin bestehendem V. a. ein spinales Liquorleck oder eine Liquorfistel eine digitale Subtraktionsmyelographie sowie eine Postmyelocomputertomographie durchgeführt. In diesen Untersuchungen konnten jedoch kein Liquorleck oder Liquorfistel nachgewiesen werden. Im Rahmen der Lumbalpunktion zur Durchführung der Myelographie wurde Liquor mit nun normwertigen Demenz- und Destruktionsmarkern gewonnen. Es wurde eine Vorstellung zur Zweitmeinung in einem hinsichtlich subokzipitaler und spinaler Liquorleckdetektion spezialisierten Zentrum gebahnt.

## Diskussion

Beim „Brain-sagging-dementia“-Syndrom (BSD) handelt es sich um eine seltene, potenziell reversible Demenzursache, die durch eine spontane intrakranielle Hypotension verursacht wird und in der aktuellen Literatur zunehmende Berücksichtigung findet [1–3]. Bereits 2002 beschrieben Hong et al. eine Kasuistik eines Mannes mittleren Alters mit spontaner intrakranieller Hypotension und kognitiven sowie behavioralen Auffälligkeiten, die unter spezifischen Therapiemaßnahmen reversibel waren [4]. Klinisch ist das Syndrom meist durch eine schleichend progrediente Entwicklung kognitiver Defizite und behavioraler Auffälligkeiten charakterisiert, die aufgrund erheblicher symptomatischer Überlappungen an eine behaviorale Variante einer frontotemporalen Demenz (bvFTD) denken lassen [5]. Als ursächlich angenommen werden mechanische Effekte auf frontale Kortexstrukturen und frontale Netzwerke durch das „brain sagging“ [1, 5]. Hierbei werden auch tierexperimentell Verhaltensveränderungen insbesondere im sozialen Kontext bei Alteration des dorsalen tegmentalen Graus oder des Thalamus beschrieben [6–8]. Die Erkrankung manifestiert sich meist in der 6. Lebensdekade und betrifft überwiegend männliche Patienten (Verhältnis Männer:Frauen = 4:1; [5]). Die Mehrzahl der Patienten (89 %) leidet unter orthostatischem Kopfschmerz, wobei die orthostatische Komponente auch fehlen kann [5]. Auch eine Kopfschmerzzunahme im Tagesverlauf wird beschrieben [5]. Ein bildmorphologisch nachweisbares „brain sagging“ in Abwesenheit einer frontal betonten Atrophie stellt eine wegweisende und obligatorische Befundkonstellation dar [5]. Die Therapie besteht im Verschluss eines zu detektierenden Liquorlecks, worunter 81 % der Patienten eine partielle und 67 % eine komplette Symptomremission zeigen [5]. Das Liquorleck entspricht in vielen Fällen einer zervikal bis thorakal und meist ventral gelegener Liquorfistel. Durch den langsamen Liquoraustritt können orthostatische Kopfschmerzen, erniedrigter Liquoröffnungsdruck sowie klassische bildgebende Befunde eines Liquorunterdrucksyndromes bei einer BSD fehlen [1]. Auslösend sind häufig Mikrosporne, welche es zu detektieren gilt. Aufgrund der noch geringen Bekanntheit wird dieses seltene Syndrom wahrscheinlich häufig nicht oder zu spät diagnostiziert [3].

Im vorliegenden Fall bestand bei zunächst langsam fortschreitender kognitiver Dysfunktion und entsprechendem Liquorbefund initial der Verdacht auf eine neurodegenerative Demenzerkrankung. Insbesondere das zunächst erhöhte Phospho-Tau zeigt eine hohe Spezifität für eine Alzheimer-Krankheit, sodass in den aktuellen Diagnosekriterien eine isolierte pTau-Erhöhung die Diagnose einer Alzheimer-Krankheit möglich macht [9]. Auch Abeta-40, Abeta-42 und Gesamt-Tau waren initial erhöht, normalisierten sich jedoch im Verlauf. Eine Beeinflussung der Konzentration dieser Parameter im Liquor durch pathologische intrakranielle Liquorbildungs- und Resorptionsverhältnisse erscheint daher möglich. So sind Abeta42, Tau und Phospho-Tau beispielsweise bei einem Normaldruckhydrozephalus, also einem relativen Liquorüberfluss, eher erniedrigt [10]. Nach Entlastung der größenprogredienten, potenziell lebensbedrohlichen mesenzephalen Zyste war eine eindrückliche, passagere Besserung der kognitiven und behavorialen Auffälligkeiten zu verzeichnen. Residuelle Defizite nach Entlastung der mesenzephalen Zyste sind durch die bereits seit Monaten bestehende Hernierung des Hippokampusgewebes erklärbar. Die erneute Zunahme der Defizite trotz konstanter Bildgebung weist auf eine weiter bestehende und möglicherweise langsam progrediente Pathologie bei möglicher Liquorfistel hin. Möglich erscheint im vorliegenden Fall außerdem, dass sich die mesenzephale Zyste um den thalamischen Seed durch ein Liquor-Trapping im Rahmen eines Ventilmechanismus bei wechselnder intrakranieller Hypo- und Hypertension (durch wiederholte Liquorpunktionen und das wiederholte Husten) gebildet hat. Selten wird ein Hustenreiz auch durch intrazerebrale Raumforderungen durch pathologischen Druck auf die dorsale medulläre Region provoziert [11]. Eine Verursachung der kognitiven Defizite durch die Zyste ist jedoch unwahrscheinlich, da bereits vor deren Größenprogredienz langsam progrediente kognitive Defizite und eine Herniation bestanden.

Eine kausale Therapie des „brain sagging“ ist nur durch Lokalisation und Verschluss eines Liquorlecks oder einer Liquorfistel möglich, was im vorliegenden Fall bisher nicht gelang, nun aber per Zweitmeinung angestrebt wird. Alternativ können wiederholte Blutpatches mit anschließender Kopftieflagerung durchgeführt werden, diese zeigen aber aufgrund der häufig ventralen und zervikal-thorakalen Lage der Liquorfisteln eine nur eingeschränkte Wirksamkeit. Aufgrund fehlender Therapiealternativen muss dieses Prozedere jedoch zusätzlich zu einer Bettruhe und ggf. palliativer Versorgung in Betracht gezogen werden.

## Fazit

Diese Kasuistik soll Behandler für das BSD-Syndrom als potenziell reversible Demenzursache einerseits und als potenziell lebensbedrohliche Erkrankung andererseits sensibilisieren. Die einzig Erfolg versprechende Therapie besteht in der Lokalisation des Liquorlecks bzw. einer Liquorfistel und dem Verschluss. Eine interdisziplinäre Zusammenarbeit ist unabdingbar.
